# Brain fingerprints of olfaction: a novel structural method for assessing olfactory cortical networks in health and disease

**DOI:** 10.1038/srep42534

**Published:** 2017-02-14

**Authors:** A. Fjaeldstad, H. M. Fernandes, T. J. Van Hartevelt, C. Gleesborg, A. Møller, T. Ovesen, M. L. Kringelbach

**Affiliations:** 1 Flavour Institute, Aarhus University, Aarhus, Denmark; 2 Department of Psychiatry, University of Oxford, Oxford, UK; 3 Department of Otorhinolaryngology, Regional Hospital Unit West Jutland, Holstebro, Denmark; 4 Center of Functionally Integrative Neuroscience, Aarhus University, Aarhus, Denmark; 5 Center for Music in the Brain, Aarhus University, Aarhus, Denmark; 6 Department of Nuclear Medicine & PET-Centre, Aarhus University Hospital, Aarhus, Denmark

## Abstract

Olfactory deficits are a common (often prodromal) symptom of neurodegenerative or psychiatric disorders. As such, olfaction could have great potential as an early biomarker of disease, for example using neuroimaging to investigate the breakdown of structural connectivity profile of the primary olfactory networks. We investigated the suitability for this purpose in two existing neuroimaging maps of olfactory networks. We found problems with both existing neuroimaging maps in terms of their structural connectivity to known secondary olfactory networks. Based on these findings, we were able to merge the existing maps to a new template map of olfactory networks with connections to all key secondary olfactory networks. We introduce a new method that combines diffusion tensor imaging with probabilistic tractography and pattern recognition techniques. This method can obtain comprehensive and reliable fingerprints of the structural connectivity underlying the neural processing of olfactory stimuli in normosmic adults. Combining the novel proposed method for structural fingerprinting with the template map of olfactory networks has great potential to be used for future neuroimaging investigations of olfactory function in disease. With time, the proposed method may even come to serve as structural biomarker for early detection of disease.

Affected olfactory processing has in the last decade been linked to several neurodegenerative and psychiatric diseases; hyposmia is preceding the onset of motor symptoms in Parkinson’s disease by approximately five years^1^,^2^,^3^, precedes cognitive dysfunction in Alzheimer’s disease^4^,^5^,^6^, and is closely linked to schizophrenia^7^, and depression^8^,^9^. A diminished sense of smell (hyposmia) could be a window into understanding and diagnosing several disorders of the brain. Although the link between hyposmia and neuropsychiatric and neurodegenerative diseases is still not fully understood, it is clearly linked to the underlying mechanisms of olfactory processing and the computational remodelling in disease states.

Olfactory processing lacks the thalamic gating of other sensory processing^10^. In addition, as far as is currently known, olfactory processing does not seem to share the topographical organization of stimuli comparable to other sensory modalities such as frequency encoding of auditory stimuli or spatial encoding of visual stimuli. However, non-human primate studies indicate that processing of odorant qualities such as identification, intensity, threat-level, and hedonic evaluation are highly segregated and specialized in widespread subcortical and cortical areas^11^,^12^,^13^,^14^. It is commonly accepted that the first cortical processing of olfactory stimuli takes place in the primary olfactory cortical networks (OCN) which can be found in the piriform cortex^15^. Still there is important regional variation in afferent neuron projections from the olfactory bulb to the posterior and anterior parts of piriform cortex which have been proposed to have different roles in olfaction^16^,^17^. The odour-induced activation of olfactory receptors is delivered from the olfactory bulb through parallel pathways of mitral and tufted cells (the lateral olfactory tracts) to the primary OCN. However, both structural connectivity and response latency differs between these two cell types, as tufted cells are only connected to the anterior parts of the primary OCN, where they convey distinct odour information on a wider scale of odour concentrations with shorter latency responses^18^.

Taken together, this segregation of information and seeming lack of straightforward topographical organization have led to divergent findings of different olfactory regions in the human olfactory literature, which vary depending on scanning modalities, qualities of olfactory stimuli and applied analysis methods^19^,^20^,^21^,^22^,^23^.

Structural MRI and diffusion tensor imaging (DTI) studies have investigated white and grey matter integrity loss in the main OCN and found that it is related to olfactory impairment in both patients with Parkinson’s disease and in subjects without other neural impairments^24^,^25^,^26^,^27^. The correlation between olfactory impairment and reduced fractional anisotropy in gyrus rectus^28^ and in the main OCN in Parkinson’s patients^24^ is indicative of a conditional change in olfactory network connectivity. Central hyposmia has a key role as an early prodromal symptom and potential marker of disease progression in this heterogeneous list of disorders, ranging from Parkinson’s and Alzheimer’s disease to depression, and schizophrenia^29^,^30^. In anhedonic states such as depression, olfactory stimuli are often perceived as less pleasant and more aversive^31^. This mechanism is perhaps conveyed through perturbed processing in secondary olfactory areas. As the pleasant odours are processed in a specific network, which includes the medial orbitofrontal cortex (OFC), while unpleasant odours induce changed activity in lateral OFC and hippocampus, changes in the OCN of patients may quite specific for their disease^32^,^33^,^34^,^35^.

Human neuroimaging methods lack the spatial specificity of tract tracing methods that can be used in animals and have not had great success in localizing the olfactory cortical areas^36^. Still, one approach for parcellation of the olfactory areas of interest has been to extrapolate data from functional olfactory neuroimaging studies to localize areas of olfactory activation using an activation likelihood estimation (ALE) analysis^37^, a popular tool for meta-analysis of neuroimaging data. A recent meta-analysis of 40 olfactory fMRI and PET studies applied ALE analysis in order to determine the statistical location of main human OC. This careful study provided coordinates for a probabilistic map of olfactory activity, by statistically combining all published data from functional olfactory neuroimaging^38^. Olfactory regions of interest were created by converting voxels with significant olfactory activity (defined as a t-value > 50%) into anatomical masks. This statistical functional localization of the human functional olfactory cortex networks (fOCN) was made available online and has subsequently been used as a reference for several olfactory neuroimaging studies^39^,^40^,^41^. However, the use of these brain coordinates for subsequent neuroimaging studies has its limitations; e.g. fast olfactory processing cannot be adequately examined in detail with the relatively low temporal resolution of fMRI^42^ and especially given the temporal scale PET (on the scale of minutes) included in this meta analysis. Furthermore, it is somewhat surprising to find that while the identified statistical localization of the human primary OCN in this paper includes the posterior piriform cortex and parts of the amygdala, it also contains regions without direct inputs from the olfactory bulb (e.g. hippocampus, parahippocampus, and putamen) as well as voxels of white matter and CSF. As the primary OCN is defined as cortical regions receiving direct input from the olfactory bulb, it may not be ideal to include secondary areas in a parcellation of primary OCN^43^,^44^,^45^,^46^,^47^.

In addition, the anterior OCN areas of the piriform cortex receive information from tufted cells and are not included in the fOCN map, which thus can filter out important information of olfactory processing. The cortical areas involved in processing of olfactory stimuli are functionally segregated, where the anterior piriform cortex exhibits important functions, e.g. in categorizing odours^48^, behavioural odour responses^49^, and odour imagery^17^.

Alternatively, the main OCN can be defined using existing anatomical knowledge as for example used in the widely-used AAL template^43^, where the authors described the structural OCN (sOCN) by isolating the olfactory cortices as defined by Dejerine^50^ and including the olfactory tubercle (on the caudal side of the gyrus rectus within the two branches of the fourth frontal sulcus), as well as Broca’s olfactory cortex located under the corpus callosum genu^51^. Still, such a structural map taken from only one individual is likely to be too coarse to adequately describe the complete structural anatomy of the main OCN.

Characterization of functional segregation can be examined by analyzing patterns of anatomical connectivity^52^.This method has been widely applied, where inter- and intra-regional structural connectivity is analyzed to define functional cortical sub-regions in the thalamus^53^, the temporal pole^54^, anterior temporal regions^55^, middle temporal gyrus^56^, cingulate cortex^57^, as well as for language processing^58^ and target selection in DBS^59^. Thus, investigating structural olfactory connections may add valuable information to our understanding of normal olfactory processing.

In this paper, we developed a novel method for fingerprinting the anatomical connectivity of the functional OCN map (fOCN) and the structural OCN map (sOCN). This allowed us to identify the structural connectivity networks linking the OCN to key secondary olfactory regions of the brain in healthy normosmic participants. Based on the findings we aimed to create a common template that can be used in future neuroimaging studies of olfactory function in health and disease.

## Results

A global, structural connectivity matrix was created using DTI data and probabilistic tractography, which formed the basis for the olfactory connectivity fingerprints and the identification of primary olfactory sub-regions.

An initial step before applying probabilistic tractography to the fOCN region was to determine the exact location and extent of overlapping regions within the AAL parcellation. We found that the fOCN could be divided into two: one part overlapping regions of primary olfactory processing (posterior piriform cortex and amygdala), and one part overlapping regions not receiving direct input from the olfactory bulb, hence not per definition primary OCN (hippocampus, parahippocampus, putamen, white matter and CSF). This was confirmed by overlaying the fOCN template with the Harvard-Oxford atlas^60^ that was used for defining other brain regions in the meta-analysis where the fOCN was defined^38^. The presence of white matter was also confirmed by visual inspection of the fOCN in all participants’ subject space T1 MRI images.

For interpretation of the olfactory connectivity fingerprints, it is important to emphasize that the overlapping fOCN voxels of the areas are subtracted from the normal AAL parcellation of these regions (Table 1). Not doing so would result in calculating a connectivity measure from a region to itself, which would be misleading and potentially confounding. Due to the removal of small overlapping parts of the hippocampus, parahippocampus, and putamen in the connectivity analysis of the fOCN, there is a small difference in the parcellation used in the fOCN and sOCN connectivity fingerprint.

### Olfactory connectivity fingerprint of the fOCN

**Figure 1:**
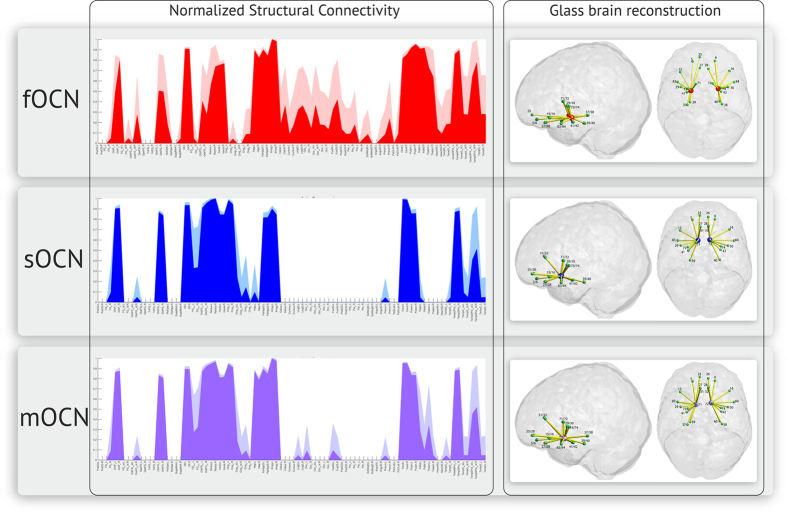
Structural connectivity fingerprint of the fOCN, sOCN and mOCN respectively. The normalized structural connectivity plots display the mean connectivity and standard deviation (lighter colour), across all subjects, from the OCN seed of each template to all remaining areas in the AAL brain parcellation. The normalized connectivity measures are used to create a glass brain reconstruction of the structural olfactory connectivity network. The thickness of the edges (in yellow) indicates the average connection strength across all subjects. The green spheres represent the centre of gravity of each area involved in the secondary olfactory processing (for AAL area numbers, see Table 3).

The anatomical connectivity patterns between the fOCs and the 90 regions of the AAL parcellation are shown in Fig. 1. The two fOCN maps (one in each hemisphere) were connected to several deep and cortical structures (Table 2), and partly situated within the ipsilateral amygdala. Across all subjects, these two maps have ipsilateral connections to the sOCN, hippocampus, parahippocampal gyrus, pallidum, superior temporal poles, amygdala (remaining part), and thalamus (temporal, occipital, and the posterior parietal parts). The right fOCN map has unilateral connections to the caudate nucleus and the superior frontal gyrus. The fOCN maps were only sporadically connected to the OFC across subjects.

### Olfactory connectivity fingerprint of the sOCN

The anatomical connectivity patterns between the sOCN and the other 89 regions of the AAL parcellation are shown in Fig. 1. The two fOCN maps (in each hemisphere) are connected to several deep and cortical structures (Table 2), which were not identical to connections of the sOCN. Across all subjects, the two sOCN maps have ipsilateral connections to the temporal pole, amygdala, caudate nucleus, parahippocampal gyrus, anterior cingulum and several sub regions of the orbitofrontal cortex.

### Connectivity-based parcellation of sub-regions within the merged olfactory cortex network (mOCN)

**Figure 2:**
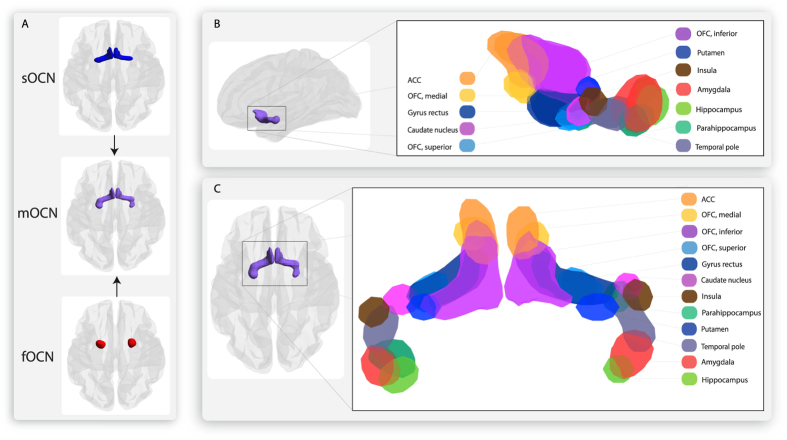
New merged olfactory cortex network (mOCN) and sub-regions. (**A**) Contributions from the sOCN and fOCN to the mOCN cluster. (**B**) Lateral view. (**C**) View from below. There is a different connectivity profile in the anterior part (derived from the sOCN) and the posterior part (Derived from parts of the fOCN). To discriminate which connections of mOCN are derived from the original sOCN and fOCN clusters, please compare Fig. 2A with Table 2. OFC: Orbitofrontal cortex. ACC: Anterior cingulate cortex.

As the original fOCN map contains regions, which are not part of the main OCN, these parts of the fOCN overlapping hippocampus, parahippocampus, and putamen were taken away (as well as non-grey matter areas), leaving only primary OCN areas in the map which was then combined with sOCN to produce the mOCN map. The differences in connectivity to secondary olfactory areas were segregated across the combined sOCN and fOCN: the anterior part of the mOCN, representing the piriform cortex, was strongly connected to the OFC, gyrus rectus and the ACC. The posterior part of the mOCN, representing the amygdala part of fOCN, was strongly connected to hippocampus, parahippocampus and the remaining part of amygdala. As predicted from the important link between memory and olfactory stimuli, removing the non-primary OC voxels covering hippocampus and parahippocampus did not eliminate the structural connectivity to these areas in the mOCN. The anatomical sub-regions of the merged mOCN (merged from sOCN and parts of fOCN) and the connectivity to other regions of the AAL parcellation are shown in Fig. 2.

## Discussion

We have developed a novel method for fingerprinting of structural olfactory connectivity which is based on our previous work^59^. This novel method was used to investigate two existing functional and structural maps of olfactory cortical networks (fOCN and sOCN). In a young normosmic population without signs of disorder known to affect olfactory function and processing, we tested the suitability of using such maps as markers of olfactory function in neuroimaging data, as both the fOCN^39^,^40^,^41^ and the sOCN^61^,^62^,^63^,^64^ are already widely used in olfactory research. We found that while these maps have been carefully constructed and clearly useful, neither of the existing maps describe the existing known olfactory connectivity sufficiently well to be used as routine markers of olfactory neuroimaging activity. Instead, we constructed a merged map - mOCN - that does contain the expected structural connectivity and could be used in future studies; potentially as a neuroimaging biomarker to describe the early structural changes in connectivity found in disease states such as Parkinson’s disease.

Specifically, our new method uses probabilistic tractography and anatomical parcellation to investigate inter- and intra-regional differences and characteristics of olfactory structural connectivity, thereby combining methods that have proven to have high reliability and validity in recent studies on brain parcellation^54^,^55^,^58^,^59^. We used two well-established OCN maps from the literature^38^,^43^ and extracted the connectivity fingerprints in a normosmic adult population.

Anatomically, we showed that the regions within the fOCN map overlap with parts of the posterior piriform cortex, amygdala, hippocampus, parahippocampus, putamen, white matter and CSF.

In terms of connectivity, the fOCN is connected ipsilaterally to the other parts of the amygdala, piriform cortex, and putamen, as well as to hippocampus, parahippocampal gyrus, pallidum, the temporal poles, insula, caudate nucleus and the thalamus. In the right fOCN a connection to the ipsilateral right superior frontal gyrus is found in all subjects. But surprisingly we did find connectivity to the orbitofrontal cortex, which is a well-established secondary olfactory region. In contrast, we found that the sOCN is connected ipsilaterally to several parts of the orbitofrontal cortex as well as the amygdala, gyrus rectus, insula, anterior cingulate cortex, parahippocampal gyrus, caudate nucleus, putamen, the temporal pole and the contralateral OCN.

The two maps for the OCN are defined by widely different methods: using anatomical methods for localising the sOCN and a statistical analysis of PET and fMRI activity related to olfactory stimuli for fOCN. Thus, it is not surprising that there are differences in the underlying OCN maps and their connectivity. Both approaches clearly have several strengths and we therefore combined them in a merged map, which has the advantage of including all of the important connections to secondary olfactory areas. This new mOCN map is thus applicable to structural and functional neuroimaging modalities, which is vital for further developing our understanding of the olfactory function.

This is of major relevance given how the sense of smell guides us in many aspects of life: from picking the fresher fish at the market, selecting our partner^65^, to seeking pleasure from food and beverages when smell and taste merge in flavour perception^66^. The importance of smell is driven by the remarkable connectivity from the olfactory bulb and primary olfactory cortical networks (OCN) to important brain regions controlling pleasure, emotions, and memory^67^,^68^,^69^. As the OCN in normosmics may be affected by age and other factors known to modulate olfactory function and perception, a thoroughly matched control group will be a prerequisite for analysing changes in structural olfactory connectivity in any future studies. As such, having a neuroimaging map that can guide us in assessing olfactory brain processing and connectivity in health and disease could help with gaining a better understanding of anhedonia^70^ – and perhaps even be used as a potential early biomarker of disease.

### Discussion of the structural connectivity of the sOCN

The main regions connected to the sOCN are the piriform cortex, OFC and anterior cingulate cortex as discussed in the following.

#### Piriform cortex

The sOCN is defined in the widely-used AAL template, in which the piriform cortex constitutes a large part of the OC parcellation, but also including the olfactory tubercle^43^. The importance of the piriform cortex as a central hub of the primary OC has been established by anatomical studies^44^,^45^,^46^,^47^,^71^,^72^, where the three-layered cortical surface represents a structural relic of olfactory importance through cortical evolution^73^. Variations in anatomy, physiology and function suggests that that piriform cortex consists of two different sections, the posterior and anterior piriform cortex^74^. While a small portion of the posterior piriform cortex was included in both the sOCN and fOCN maps, the anterior piriform cortex is only included in the sOCN map and not the fOCN map. A functional connectivity between the bilateral anterior piriform cortices have previously been described^75^, which is consistent with our structural findings.

#### OFC

There is strong connectivity to one of the most well described areas of secondary olfactory processing is the OFC, which was only present in the sOCN, as the connectivity was exclusive to the anterior piriform cortex, see Fig. 2. The link between olfaction and the frontal cortex was established in the 1940 s on animal models^76^, which was later reproduced with the use of evoked potentials^77^, and later in human studies^78^ A direct anatomic connection between the OC and OFC was later found in monkeys using axonal tracers^45^. In humans, the OFC is key for higher order processing of olfactory input and multisensory integration^32^,^33^,^36^,^68^,^78^,^79^ including hedonic processing of odours^13^.

#### Anterior cingulate cortex and caudate nucleus

The sOCN display consistent structural connectivity across all participants to regions of the anterior cingulate cortex (ACC). Along with the caudate nucleus, regions of the ACC have been recognized as key for correct odour recognition in several studies, including a recent study on functional connectivity in olfactory memory tasks^80^. This is in line with the key functions of the caudate nucleus in associative learning^81^, reward^82^ and more general hedonic processing of the ACC^13^, including its role in reward-based decision making^83^,^84^.

### Olfactory anatomy and structural connectivity of the fOCN

The fOCN map includes much of the known olfactory brain regions as shown below.

#### Amygdala

A substantial part of the fOCN is located within the amygdala, when merged into the AAL template. As the amygdala receives olfactory input directly from the olfactory bulb, this is defined as a part of the main OCN^45^. The processing within the amygdala is characterized by subdivisions and nuclei (laterobasal, centromedial, and superficial) with a high degree of inter-connectivity and will not be further described in this paper (see review by Roy *et al*.^85^). The role of amygdala in olfactory processing is well established; though it is specifically involved in hedonic valence and emotional behaviour with a high sensitivity to olfactory stimuli^86^,^87^,^88^, activation of amygdala is elicited by all odours, regardless of hedonic value^46^.

#### Hippocampus

In all participants, the fOCN was found to have high degree of connectivity to the hippocampus; connections which are thought to play a major role in learning and memory (see recent work by McDonald and Mott^89^). However, the fOCN map does include a small part of the hippocampus parcellation, why the structural connectivity profile of the fOCN to this area is not highly reliable. Still, the subsequent results of using our novel mOCN map, adding primary olfactory regions from the fOCN, the connectivity analysis of the amygdala part of the fOCN still contained high degree of connectivity to the hippocampus. This is not surprising given to the close relation between odour and memory.

#### Pallidum

Though many regions are involved in the hedonic circuitry, the ventral pallidum is the only area where a localized neural damage has been shown to eliminate the capacity for a positive hedonic reaction^90^. In rodents, this localized damage to the ventral pallidum can even alter normal liking-behaviour for sweet stimuli to a repulsive-behaviour identical to responses observed when presented to noxious stimuli^91^. Apart from the hedonic component of ‘liking’, the ventral pallidum is also involved with the motivational hedonic component of ‘wanting’^92^, The ‘wanting’ component of hedonic processing is represented by a different subset of neurotransmitters and hedonic circuitry^90^.

Across all subjects, the fOCN was structurally connected to the pallidum. However, when focusing on the established grey matter regions of the fOCN map, the structural connection to the pallidum disappears, see fOCN (grey) in Table 2. This may be due to the white matter voxels included in the original fOCN map, as identified with the Harvard-Oxford-atlas (Table 1) and by visual inspection of the fOCN in all participants’ subject space T1 MRI images. As a highly interconnected part of the basal ganglia, the ventral pallidum is a hedonic hotspot which is one of the key regions for the processing of liking and pleasure^93^. However, the key neural circuitries of the pallidum are structural connections from the hippocampus and nucleus accumbens, and not the amygdala^94^. The role for pallidum in the hedonic processing of odours^95^ could likely to be of an intermediate connecting hub, such as the hippocampus.

#### Thalamus

Although the olfactory system is unique, as sensory input is not undergoing thalamic relay^10^,^47^, the fOCN was connected to thalamus which is not surprising given that DTI does not measure the directionality of fibres. Like for the connections to pallidum, these connections disappeared when applying a more strict focus on the grey matter regions of the fOCN (Table 1). Though the mediodorsal thalamic nucleus has been suggested to have a role in olfactory attention^96^, we find no common structural connectivity between the grey matter OC and thalamus. However, this regulation may be mediated by the extensive connections between other regions such as the OFC and the mediodorsal thalamic nucleus^97^.

### Creating a restricted grey matter-fOCN

As the fOCN map contains voxels of white matter classified using the AAL and Harvard-Oxford atlases (Table 1) and by visual inspection across all subjects, an accurate measure of structural connectivity becomes challenging. The structural connectivity between brain regions is constructed by measuring the strength in fibres connecting the different grey matter regions in the parcellation. If voxels of white matter are included in the parcellation, the connectivity between seed and target region of these fibres will disappear and become an incorrect measure of connectivity from the white matter voxel to both the seed and target regions. Though the fOCN parcellation was not created for the purpose of measuring structural connectivity, important information of functional activation can still be extrapolated from the fOCN template. Thus, a cleansed fOCN template containing only grey matter regions (based on the AAL atlas) was produced in order to compute a more accurate connectivity matrix (the template ‘fOCN (grey)’) (Table 2).

### Olfactory structural connectivity shared by the fOCN and the sOCN

#### Insula

The insula was an integrated part of both the sOCN and fOCN across all subjects, which is in accordance with earlier studies, where the insular input to the OC were found to be most pronounced in the posterior piriform cortex^98^. These neurons in the posterior piriform cortex have been shown to respond selectively to taste stimuli, representing a segregated function of chemosensory convergence in this part of the OC^99^. Furthermore, the insula is key in integrating the subjective awareness of a widespread list of other bodily sensations, such temperature, pain, hunger, thirst, touch, sound, and of course taste.

#### Putamen

The fOCN and sOCN were structurally connected to putamen. A small area of fOCN overlapped with the putamen, which may affect this connectivity measure. However, after subtracting these overlapping voxels from the fOCN, the structural connectivity persisted in the fOCN (grey) (Table 2). Putamen plays a role in implicit learning^100^, but is also involved in dopamine reward responses during food consumption^101^ where it is believed to modulate the perception of contempt and disgust^102^.

#### The temporal pole

The temporal poles were structurally connected to both sOCN and fOCN in all subjects. This highly interconnected area is a well-known recipient of projections from amygdala, OFC, piriform cortex and insula, and is proposed to play a role in coupling emotional responses to highly processes sensory stimuli^103^. Patients with injuries in the right temporal pole perform poorly in odour recognition tests^104^,^105^, indicating the importance of this area for retention of namable odours^106^. Apart from memory, only one study has described involvement of the temporal poles in other aspects of olfactory processing; in this study of patients with olfactory dysfunction after head trauma, an intact temporal pole was related to the occurrence of distorted olfactory processing (parosmia) after injury^107^.

#### Parahippocampus

The parahippocampal gyrus was connected to both the sOCN and fOCN ipsilaterally on both sides. Like the hippocampus and the temporal pole, this region is closely related to olfactory memory^80^. It has been shown that specific odours linked to autobiographical memory can elicit widespread activations of amygdala and parahippocampal cortex and affect breathing patterns^108^. For a comprehensive review on the neural basis on odour memory, see ref. 109.

### Sub-parcellation of the mOCN

As expected from the dissimilarity of structural connectivity profiles between the sOCN and the fOCN, the sub-parcellation of the mOCN displays variances between the anterior and posterior parts. Even within the single areas, such as the piriform cortex, the connectivity fingerprint differs substantially. These differences in connectivity are in concordance with previous studies^46^,^74^,^75^. In a recent Neuron paper, the authors identified spatiotemporal activity patterns unique to the anterior piriform, linking this specific part of the OC to activity in the OFC both before and after olfactory stimuli, while the posterior piriform cortex showed activation related to odour-specific prediction models^16^. These differences are consistent with the findings of our study, along with the connectivity to key secondary areas such as the OFC, hippocampus and anterior cingulate cortex (Table 2 and Fig. 1).

## Conclusion

The differences in connectivity profiles between sOCN and fOCN underline the importance of specificity of OCN regions for different odour related processing, resulting in segregated activation of cortical and subcortical areas by olfactory input. The lack of inclusion of parts of the main OCN in neuroimaging studies can significantly alter the integration and segregation of olfactory processing. We integrated the two existing OCN maps to a common merged OCN (mOCN) encapsulating the relevant areas of cortical and sub-cortical olfactory processing. This can be used as a map in future neuroimaging studies; for functional as well as structural studies, potentially generating a unified field of human neuroimaging olfactory research. As olfactory stimuli can generate widespread sensory and hedonic processing, this novel approach for investigating the brain connectivity profile of olfactory processing could become a highly useful tool for characterising hedonic processing in health and disease; perhaps even as an early biomarker.

## Methods

To map the connectivity between brain regions of interest, we applied probabilistic tractography to obtain comprehensive whole brain neural networks based on diffusion tensor imaging (DTI). This method can utilize the underlying measures of fractional anisotropy, local level of mean diffusivity, radial diffusivity or axial diffusivity^110^,^111^,^112^. We redesigned a recently proposed algorithm for targeting specific structural networks in individuals prior to placement of deep brain stimulation electrodes in patients with chronic pain^59^.

### Study population

Data from 16 subjectively normosmic, right handed, healthy participants between 20 and 29 years of age were analysed (Table 3). All participants underwent nasal endoscopic examination, screening for cognitive impairment (Mini-Mental State Examination (MMSE)), depression (Major Depression Inventory (MDI)) and sinonasal symptoms (Sino-Nasal Outcome Test (SNOT-22)). Participants reported having no major issues with smell in the SNOT-22 questionnaire. Olfactory function was evaluated using the Sniffin’ Sticks 12-Identification teat and found within normal range^113^. Subjective assessments of olfactory abilities correlate poorly with actual olfactory performance^114^. Thus, testing these potential confounders of olfactory function and processing, along with nasal patency and olfactory function is fundamental for the assessment of healthy cortical olfactory networks.

All study participants received oral and written information and signed an informed consent prior to participation. The study was performed in accordance with the Declaration of Helsinki for medical research and approved by the Research Ethics Committee of the Central Denmark Region (De Videnskabsetiske Komitéer for Region Midtjylland).

### Image acquisition

Structural MRI and DTI scans were performed with a 3 T Siemens Skyra scanner with a 32-channel head coil in Aarhus, Denmark. The structural T1 Scans had a voxel size of 1 × 1 × 1 mm, slice thickness of 1 mm, matrix size 256 × 256, FoV 256 × 256, repetition time 2300 ms, echo time 3.8 ms, Pixel bandwidth 170 Hz/Px, and a Flip angle of 8˚. The DTI Echo Planar Imaging sequence had a voxel size of 1.98 × 1.98 × 2 mm, slice thickness of 2 mm, matrix size 106 × 106, FoV 210 × 210, repetition time 9000 ms, echo time 84 ms, *b*-value 1500 s/mm^2^, diffusion directions 62 (9 *b*0 scans interspersed throughout the scans, every 8 volumes), two phase encoding directions (anterior-posterior and posterior-anterior), pixel bandwidth 1745 Hz/Px, and a flip angle of 90°.

### Fingerprinting structural connectivity

The estimation of structural connectivity involves the use of a probabilistic tractography algorithm, which generates a connectivity distribution from all seed voxels, as defined by the anatomical template selected. We have used the term ‘fingerprint’ to characterise a robust network signature of connectivity patterns. Constructing fingerprints of structural connectivity for each participant’s individual anatomical regions and fOCN was done in five steps: defining individual parcellation of brain regions from the MNI template (90 AAL regions), identifying individual regions of interest from MNI coordinates by connectivity analysis, compensation for orbital distortion on OFC tractography quality, estimating connections between regions of interest and parcellated brain regions, and lastly, subparcellating the newly defined mOCN. Each step is described here in further details.

### Defining parcellation of brain regions

The brain is parcellated into 90 subcortical and cortical regions using the Automated Anatomical Labelling (AAL) brain atlas^43^. We used the FLIRT tool (FMRIB, Oxford, UK)^115^ to linearly co-register the standard ICBM152 in MNI space^116^ into the T1-weighted structural image, by using an geometric registration and a nearest-neighbor interpolation.

The resulting transformation matrix was subsequently concatenated with the previously created T1 to DTI native space transformation matrix, allowing a direct co-registration of the AAL template in MNI space to the diffusion MRI native space. This last transformation was accomplished using a nearest-neighbor interpolation method to ensure that discrete labeling values were preserved.

### Parcellation identification

The structural connectivity of two regions was measured and compared, the sOCN^43^ and the fOCN^117^. The MNI-coordinates for the fOCN were added to the 90 regions of the AAL template and the overlapping areas of this region of functional olfactory activation were subtracted from the AAL template.

### Compensating for orbital distortion on orbitofrontal tractography quality

The OFC is known to be highly susceptible to distortions (because of the junction between tissues of differing magnetic susceptibility). A weighted estimation of accuracy likelihood was made for the dual phase encoding direction, reconstructing a single set of data with significantly reduced distortions^118^.

### Estimating fingerprints

**Figure 3:**
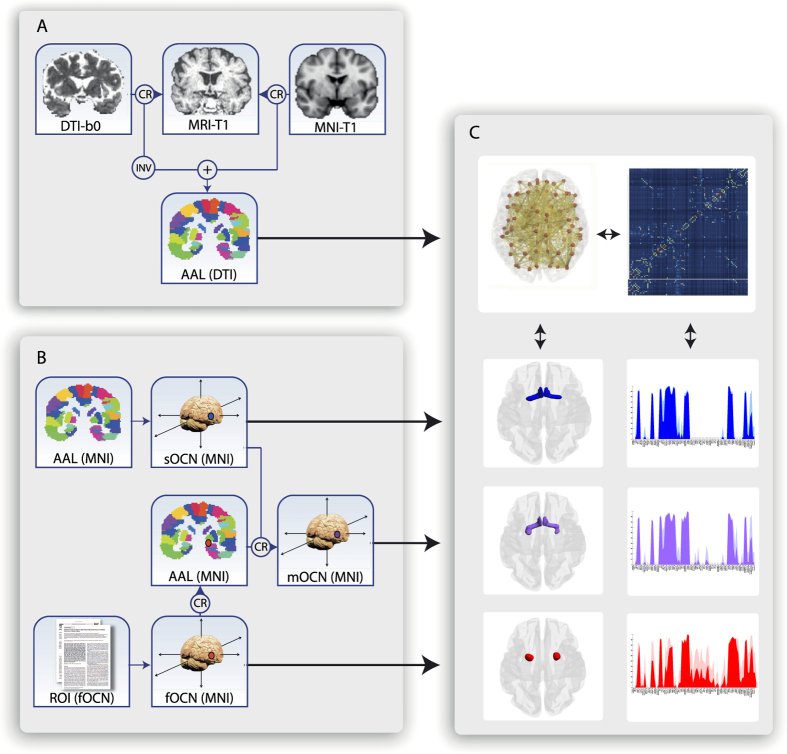
Processing pipeline. (**A**) The AAL brain parcellation was coregistered (CR) to the native space of all subjects. This was done by using a 12-degree linear registration technique (FLIRT) from both AAL and DTI to T1 space, inversion of the DTI-to-T1 transformation matrix, and subsequent combination of the two transformation matrices. (**B**) MNI coordinates for olfactory regions of interest were included from the AAL brain template (Structural olfactory cortex (sOCN)) and the statistical functional localization of the human olfactory cortex (fOCN)^38^. In each participant’s native space, a brain parcellation template based on the strategy used in Fig. 3A for automated anatomical labelling of activation (AAL)^43^ was registered allowing probabilistic tractography between fOCN, sOCN, and AAL regions. (**C**) A fingerprint of each OCN was estimated based on the underlying structural connectivity with the other anatomical regions (see Fig. 2). The parts of AAL regions overlapping the fOCN were removed from fingerprint analysis, to avoid biases in connectivity measures. CR: co-registration; INV: Inversion; + : Merge of images.

In FSL (FMRIB Software Library, Oxford, version 5.0, www.fmrib.ox.ac.uk/fsl/) we used the FDT toolbox (FMRIB’s Diffusion Toolbox) to run the multiple processing stages (Fig. 3), including FSL’s brain extraction tool (BET), BEDPOSTX, and PROBTRACKX (See FMRIB website for technical details). The pre-processing steps involved correction for eddy current gradients induced by the magnetic field and movements of the subject’s head, using the EDDY algorithm. A model for crossing fibres within each voxel of the brain was applied using a Markov Chain Monte Carlo sampling algorithm in order to calculate distributions on diffusion parameters and estimate the fibre direction probability distribution at each brain voxel^119^. With a maximum of two directions, we used an automatic estimation of fibre directions within each voxel, in order to improve the tracking sensitivity of non-dominant fibre populations in the human brain^120^.

After defining brain boundaries using the brain extraction tool, probabilistic tractography was applied to estimate the connectivity probability for each voxel, using a sampling of 5000 streamline fibres per voxel. A connectivity measure from each seed voxel *i* to another voxel *j* was defined by the proportion of fibres passing through voxel *i* that reach voxel *j*^120^. By adding *n* voxels within a given anatomical region, the connectivity measure was extended from the voxel level to the region level with 5000*n fibres. The connectivity probability P_*ij*_ from region *i* to region *j* was calculated as the number of sampled fibres in region *i* that connect the two regions divided by 5000*n, where *n* is the number of voxels in region *i*^59^. To reduce the number of false positive connections, a threshold of the connectivity probability value was set to only include connections with a minimum value of 1 per cent of the maximum number of streamlines from region *i* to region *j*.

The connectivity probabilities from the fOCN to each of the 90 AAL regions were calculated. As the sOCN is defined as the olfactory AAL region, the connectivity probabilities from the sOCN to each of the 89 remaining AAL regions were calculated. We implemented this calculation of regional connectivity probability using in-house Perl scripts. Regional connectivity was normalized using the number of voxels within each region. The structural connectivity was calculated as the weight of the edge connecting two regions. As mentioned earlier, this weight indicates the number of streamlines (out of maximum of 5000) from region *i* reaching region *j*. For each subject, connectivity matrices were constructed, representing the structural connectivity networks across the brain for the left and right fOCN and sOCN.

### Estimating primary olfactory sub-regions connected with secondary olfactory areas

A connectivity matrix was constructed in order to create a sub-parcellation of the fOCN and sOCN based on their connectivity to key secondary olfactory areas (Table 2). By identifying the seeds of connections to the secondary olfactory regions within both the sOCN and the primary olfactory part of the fOCN, the mOCN template was created - a core primary olfactory template encapsulating connections to all key secondary olfactory areas. Each possible seed voxel within the brain parcellation connected to either sOCN or fOCN was analysed as a potential connection to the mOCN by computing 5000 samples from the connectivity distribution to target voxels in the mOCN. A fixed threshold was applied to reduce false-positive connections, while preserving sufficient sensitivity for important connections; if 25% or more fibres (1250 of 5000 fibres)^121^ of a given voxel were connected to the mOCN, this voxel was classified as a seed connected to the mOCN and binarised^53^.

By applying probabilistic tractography analysis, it was possible to combine the connectivity of individual subjects to a group level connectivity, in order to calculate the likelihood of connections across individuals in the mOCN subparcellation. To focus on consistent connections across individuals, a threshold was applied to display only connections present in at least 50% of subjects^54^.

## Additional Information

**How to cite this article**: Fjaeldstad, A. *et al*. Brain fingerprints of olfaction: a novel structural method for assessing olfactory cortical networks in health and disease. *Sci. Rep.* **7**, 42534; doi: 10.1038/srep42534 (2017).

**Publisher's note:** Springer Nature remains neutral with regard to jurisdictional claims in published maps and institutional affiliations.

## Figures and Tables

**Table 1:** Brain areas included in fOCN.

Brain region	Right side		Left side	
	AAL	Harvard Oxford	AAL	Harvard Oxford
**Primary olfactory areas**				
Piriform cortices	7%	*	8%	*
Amygdala	27%	24%	37%	45**%**
**Secondary olfactory areas**				
Putamen	17%	17%	9%	6**%**
Pallidum	0%	3%	0%	1**%**
Parahippocampus	0%	0%	7%	3**%**
Hippocampus	< 1%	0%	6%	< 1**%**
Orbitofrontal Cortex	0%	5%	0%	0%
**Atlas-definition differences**				
Un-named grey matter areas	—	~37%	—	35**%**
White matter**	—	~15%	—	12%
Not contained in atlas**	50**%**	—	32**%**	—

To investigate the percentage of overlap of the fOCN on non-primary olfactory areas, the fOCN was added to both the AAL atlas and the Harvard-Oxford atlas. This conformed the initial AAL results, that the cluster contained large quantities of non-primary olfactory areas. The voxels of the fOCN cluster were subtracted from the original AAL parcellation, as any measure of connectivity to these regions would otherwise contain a nonsense measure of connection within a voxel. The fOCN did not completely overlap any AAL regions. *These areas are not defined in the Harvard-Oxford atlas, but are contained within the larger “Cerebral Cortex” parcellation. **White matter regions are only included in parcellations of the Harvard-Oxford atlas.

**Table 2:** Connectivity to anatomical areas from all olfactory cortex templates.

Anatomical area	AAL area	fOCN	fOCN (grey)	sOCN	mOCN
**Primary olfactory cortical areas**					
Piriform cortex (left)	21	100%	100%	*	*
Piriform cortex (right)	22	100%	100%	*	*
Amygdala (left)	41	100%**	100%**	100%	100%**
Amygdala (right)	42	100%**	100%**	100%	100%**
**Areas with secondary olfactory processing**					
Orbitofrontal cortex (left, superior)	5	69%	69%	100%	100%
Orbitofrontal cortex (right, superior)	6	100%	100%	100%	100%
Orbitofrontal cortex (left, inferior)	15	69%	69%	100%	100%
Orbitofrontal cortex (right, inferior)	16	69%	69%	100%	100%
Orbitofrontal cortex (left, medial)	25	56%	—	100%	100%
Orbitofrontal cortex (right, medial)	26	—	—	100%	100%
Gyrus rectus (left)	27	75%	69%	100%	100%
Gyrus rectus (left)	28	94%	94%	100%	100%
Insula (left)	29	100%	88%	100%	100%
Insula (right)	30	100%	100%	100%	100%
Anterior cingulate cortex (left)	31	—	—	100%	100%
Anterior cingulate cortex (right)	32	—	—	100%	100%
Hippocampus (left)	37	100%	100%	—	100%
Hippocampus (right)	38	100%	100%	—	100%
Parahippocampal gyrus (left)	39	100%	100%	100%	100%
Parahippocampal gyrus (right)	40	100%	100%	100%	100%
Caudate nucleus (left)	71	100%	100%	100%	100%
Caudate nucleus (right)	72	100%	100%	100%	100%
Putamen (left)	73	100%**	100%**	100%	100%
Putamen (right)	74	100%**	100%**	100%	100%
Temporal pole (left, superior)	83	100%	100%	100%	100%
Temporal pole (right, superior)	84	100%	100%	100%	100%
Temporal pole (left, middle)	87	81%	69%	50%	56%
Temporal pole (right, middle)	88	94%	94%	63%	63%
**Areas connected before removal of non-grey matter voxels**					
Calcarine fissure (right)	44	50%	—	—	—
Lingual gyrus (right)	48	50%	—	—	—
Occipital lobe (left, middle)	51	50%	—	—	—
Fusiform gyrus (right)	56	56%	—	—	—
Pallidum (left)	73	100%	—	—	—
Pallidum (right)	74	100%	—	—	—
Thalamus (left)	77	94%	—	—	—
Thalamus (right)	78	88%	—	—	—

Connectivity strength listed in percentages of subjects with structural connections between the OC template and the listed area. A threshold of 50% was applied; meaning only connections present in more than half of subjects are included. All connections are from the template to ipsilateral areas. * The sOCN and mOCN include the whole piriform cortex. ** As the fOCN templates contain parts of the amygdala and putamen, the connectivity strength is a representation of connectivity to the remaining parts of these regions.

AAL: Automated anatomical labelling atlas. fOCN: Olfactory cortical template based on functional meta-analysis. fOCN (grey): Functional olfactory cortex network template containing only defined grey matter regions. sOCN: Olfactory cortex network template based on structural data from the AAL template. mOCN: Olfactory cortical network template based on merged structural and functional data.

**Table 3:** Study population demographics.

	Study population (n = 16)	
	Mean	SD
Gender (male/female)	11/5	
Age (years)	24.75	2.54
MMSE	29.6	0.91
MDI	6.47	5.34
Handedness (n, right/left)	16/0	

Standard deviation (SD). Mini-mental state examination (MMSE). Major depression inventory (MDI).
